# Cost-Effectiveness of Five Commonly Used Prosthesis Brands for Total Knee Replacement in the UK: A Study Using the NJR Dataset

**DOI:** 10.1371/journal.pone.0150074

**Published:** 2016-03-04

**Authors:** Mark Pennington, Richard Grieve, Nick Black, Jan H. van der Meulen

**Affiliations:** 1 King’s Health Economics, Institute of Psychiatry Psychology and Neuroscience, King’s College London, London, United Kingdom; 2 Department of Health Services Research and Policy, London School of Hygiene and Tropical Medicine, Tavistock Place, London, United Kingdom; University of Exeter, UNITED KINGDOM

## Abstract

**Background:**

There is a lack of evidence on the effectiveness or cost-effectiveness of alternative brands of prosthesis for total knee replacement (TKR). We compared patient-reported outcomes, revision rates, and costs, and estimated the relative cost-effectiveness of five frequently used cemented brands of unconstrained prostheses with fixed bearings (PFC Sigma, AGC Biomet, Nexgen, Genesis 2, and Triathlon).

**Methods:**

We used data from three national databases for patients who had a TKR between 2003 and 2012, to estimate the effect of prosthesis brand on post-operative quality of life (QOL) (EQ-5D-3L) in 53 126 patients at six months. We compared TKR revision rates by brand over 10 years for 239 945 patients. We used a fully probabilistic Markov model to estimate lifetime costs and quality-adjusted life years (QALYs), incremental cost effectiveness ratios (ICERs), and the probability that each prosthesis brand is the most cost effective at alternative thresholds of willingness-to-pay for a QALY gain.

**Findings:**

Revision rates were lowest with the Nexgen and PFC Sigma (2.5% after 10 years in 70-year-old women). Average lifetime costs were lowest with the AGC Biomet (£9 538); mean post-operative QOL was highest with the Nexgen, which was the most cost-effective brand across all patient subgroups. For example, for 70-year-old men and women, the ICERs for the Nexgen compared to the AGC Biomet were £2 300 per QALY. At realistic cost per QALY thresholds (£10 000 to £30 000), the probabilities that the Nexgen is the most cost-effective brand are about 98%. These results were robust to alternative modelling assumptions.

**Conclusions:**

AGC Biomet prostheses are the least costly cemented unconstrained fixed brand for TKR but Nexgen prostheses lead to improved patient outcomes, at low additional cost. These results suggest that Nexgen should be considered as a first choice prosthesis for patients with osteoarthritis who require a TKR.

## Introduction

Over a million total knee replacements (TKR) are performed worldwide each year [1]. In 2011, the global market for knee prostheses was estimated to be $7 billion and it is projected to reach $11 billion by 2017 [2]. Most knee replacements use cement to fix the components of the prosthesis to the bone, preserve supporting ligaments (unconstrained prostheses), and have fixed tibial bearings [3].

A large number of knee prostheses have been introduced on the market and more than sixty different prosthesis brands were implanted in England and Wales in 2012 [4]. Randomised controlled trials (RCTs) have compared different types of TKR [5,6], but there are no RCTs comparing brands, and observational studies are inconclusive [7,8]. Evidence on the revision rates of the prosthesis and patients’ quality of life (QOL) according to brand is available from national joint registers [9–11]. In 2013, the National Joint Registry for England, Wales and Northern Ireland (NJR), the world’s largest database of patients who had a knee replacement, reported eight-year revision rates for the five most commonly used cemented, unconstrained brands with fixed bearings of 2.1% for PFC Sigma, 2.3% for Genesis 2, 2.4% for Nexgen, 2.8% for AGC Biomet, and 2.9% for Triathlon without case-mix adjustment [11]. A study using a sample of patients included in the NJR found that those who had received a Nexgen prosthesis had the least severe symptoms and best quality of life six months after the replacement procedure [12].

In this paper, we evaluate the relative cost-effectiveness of these five brands which together cover about 60% of the market in the UK and are among the most commonly used brands in many other countries. The study estimated lifetime cost-effectiveness for patients with osteoarthritis, separately for men and women undergoing surgery at three different age (60, 70, and 80 years). We estimated the effect of prosthesis brand on QOL using data from a national programme that collects patient-reported outcome measures (PROMs) immediately before and six months after an elective TKR in the English National Health Service (NHS), the world’s largest database of QOL outcomes following joint replacements [13].

## Methods

### Model overview

**Fig 1:**
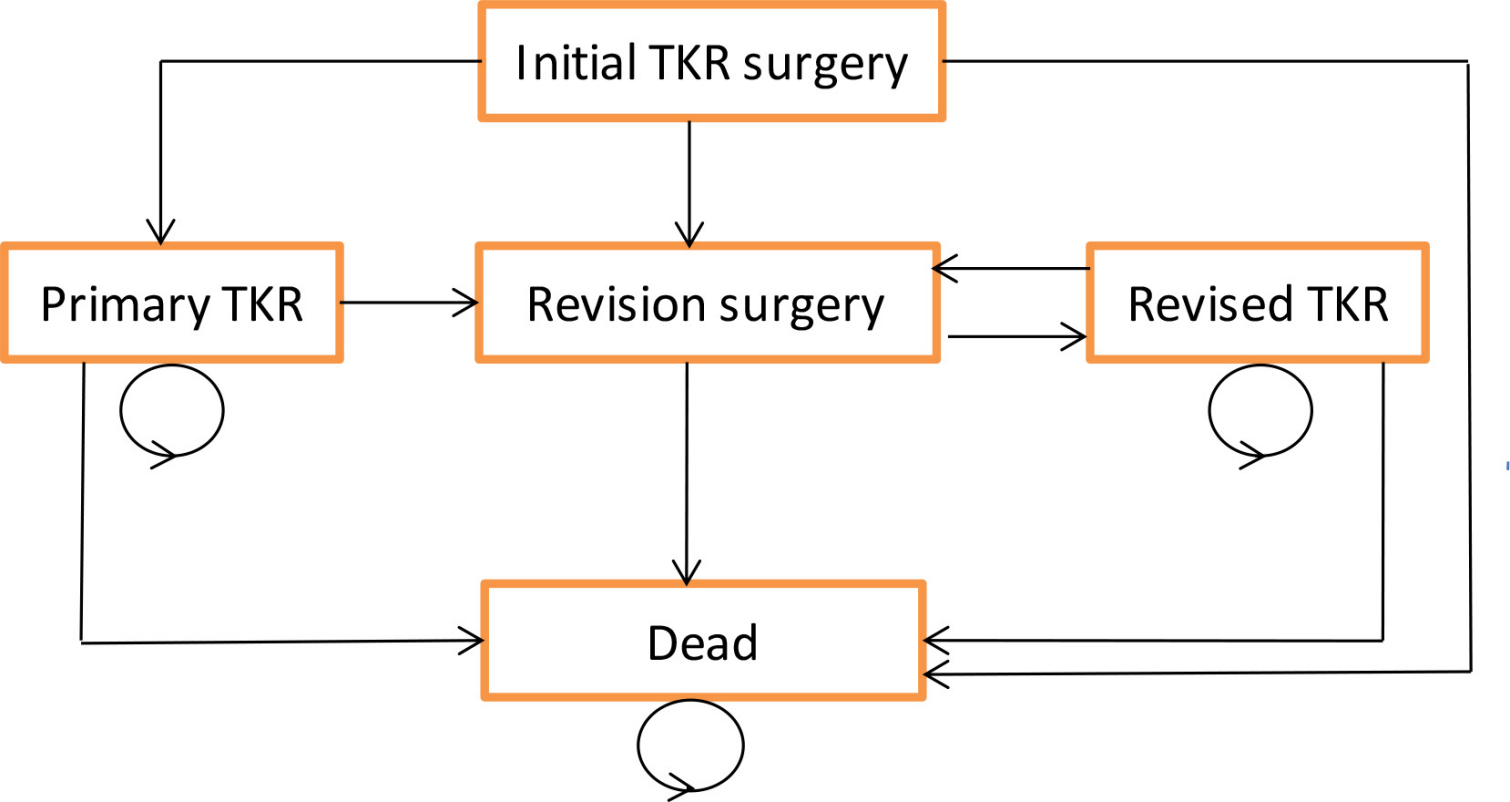
Markov model of TKR

A Markov model with a cycle length of one year was used to simulate the longevity of the knee prosthesis brands over the patients’ lifetime [14]. This model is adapted from a published evaluation of alternative types of total hip replacement, as failure of the prosthesis is the primary concern for both hip and knee replacement [15]. For each brand, costs and outcomes were estimated for a hypothetical cohort of patients who enter the model at the time of the primary TKR (Fig 1). After the primary replacement, patients face a possibility of immediate post-operative mortality, and then annual probabilities of revision of the TKR and mortality. Patients requiring revision move to the ‘Revision’ state. After successful recuperation patients then transit to the ‘Revised TKR’ state.

Time spent in health states after primary and revision TKR was weighted for QOL and summed over 45 cycles to estimate life expectancy in terms of quality-adjusted life years (QALYs). Lifetime costs from a health care perspective were calculated by summing the cost of the primary TKR and any subsequent revision procedure. We tested whether the model’s main assumptions (see Box 1) were robust to alternative assumptions in sensitivity analyses.

**Box 1. Main assumptions underlying the Markov model of the cost effectiveness analysis.**

- Patients enter the model at the time they have the TKR. The model assumes that the post-operative QOL observed at six months, applies from when the patients enter the model, and to subsequent model cycles in the TKR health state.
- The differences observed in QOL across prosthesis types six months after TKR is maintained for the lifetime, subject to the decline in QOL with increasing age, and the possibility that the TKR fails.
- The approach to estimate the effect of prosthesis type on QOL has fully addressed confounding.
- Deterioration of the prosthesis does not affect QOL adversely unless the prosthesis is revised.
- All failed prostheses are revised.
- The effect of prosthesis failure on QOL is estimated from the QOL observed before surgery in those who had a revision, and is applied for one year post revision.
- The approach to extrapolate prosthesis survival beyond the observed data accurately predicts the long term probability of prosthesis survival.
- The costs of revising the TKR are same for each prosthesis brand.
- QOL during and after revision is the same whether or not the revision was undertaken due to sepsis.

### Data sources

#### Overview

We used individual patient data from the English National PROMs Programme [13], linked to records from the NJR [11] and the English Hospital Episode Statistics (HES) database [16] to estimate the effect of TKR brand on post-operative QOL and length of stay (LOS). QOL in the primary TKR state was parameterised using *post-operative* data after primary TKR. QOL in the revision state was parameterised using *pre-operative* data after revision TKR. QOL in the revised TKR state was parameterised using *post-operative* data after revision TKR. We estimated revision rates by prosthesis brand using NJR data. Re-revision rates and mortality were estimated from HES data.

### QOL, pre-operative characteristics and prosthesis type

Data from the National PROMs Programme included patients who had an elective TKR between August 2008 and July 2012 [13]. This programme collects patient-reported comorbidities and QOL immediately before the TKR and QOL six month thereafter. The Oxford Knee Score (OKS) is a 12-item disease-specific instrument which generates scores ranging from 0 (worst health status) to 48 (best health status) [17]. The EQ-5D-3L is a generic instrument which generates health profiles using five dimensions (mobility, self-care, usual activities, pain and discomfort, anxiety and depression) and three levels (no problems, some problems, severe problems) [18]. These profiles were combined with health state preference values from the UK general population, to give EQ-5D-3L utility index scores on a scale anchored at 0 (death), and 1 (perfect health) [19].

We accessed pre-operative PROMs records for 158 799 patients aged 55 to 84 years, and subsequently included 105 637 patients whose record could be linked to NJR and HES records. The NJR provided data on prosthesis type, diagnosis (osteoarthritis or other), body mass index (BMI), and American Society of Anesthesiologists (ASA) grade [20]. HES provided data on socioeconomic deprivation derived from the patient’s postcode as the Index of Multiple Deprivation (IMD) [21].

We excluded patients who had missing data on prosthesis brand, those who had any diagnosis other than osteoarthritis or an ASA grade worse than 3; and those who received a cementless prosthesis component or a non-standard surgical procedure such as a bone-graft. The resulting sample of 53 126 patients was used to estimate LOS and QOL six months after TKR. These QOL estimates were applied to patients in the primary TKR health state in the initial cycle and each subsequent cycle. After each cycle of the Markov model we reduced QOL in each health state to reflect the effect of aging. The magnitude of the reduction increased with age (for example the reduction was 0.004 in QOL tariff at age 70) reflecting the curvilinear relationship observed in a large UK observational study [22].

For the health state representing the year in which patients had a TKR revised, QOL and LOS was taken from *pre-operative* data for 6 128 patients whose TKR was a revision. For subsequent years, QOL was taken from the 3 912 patients who had responded to outcome questionnaires *six months after the revision surgery*.

#### Rates of revision and re-revision

The annual revision rates after a primary TKR according to prosthesis brand were estimated from NJR data. We accessed 265 910 records of patients who had a primary, unilateral TKR between 1 April 2003 and 1 March 2012, who were aged between 55 and 84, who had a primary diagnosis of osteoarthritis, and who received treatment in an NHS hospital or treatment centre. Records for 239 945 patients were available for analysis after applying the same exclusion criteria we applied to the PROMs data. The data contained 3 148 linked revisions recorded in the NJR (593 PFC Sigma, 392 AGC Biomet,129 Nexgen, 112 Genesis 2, 74 Triathlon, and 1 848 other brands). Maximum observation periods varied by brand from 11.1 years for the PFC Sigma to 6.7 years for the Triathlon.

Re-revision rates were estimated from the records of 54 134 patients with a revision recorded in HES between April 1997 and March 2012, after linking initial and subsequent revisions on the same knee.

#### Mortality

Operative mortality after TKR was estimated from HES data. We did not find any difference in operative mortality across prostheses brands (p>0.4) after adjusting for potential confounders (age, sex, ASA grade, BMI, funding source and date of surgery), and therefore we applied the same probabilities of death across all prosthesis brands. Annual mortality according to age and sex was taken from general population data, after using HES data to adjust for the “healthy patient” effect, to recognise that patients who have undergone joint replacement for osteoarthritis have a lower mortality than observed in the general population [23]. The healthy patient effect was largest for older patients (relative risk of dying was 0.3 for males aged 80 in the year after surgery) and decayed exponentially to a relative risk of 1.0 over the decade following surgery.

#### Costs

All costs are reported in British pounds (1 British pound ≈ 1.60 US dollars ≈ 1.20 euros) according to 2011–12 prices. The unit cost of each prosthesis brand was taken from the average prices paid by a mid-size NHS provider (including all components and instrumentation) to reflect discounts negotiated on list prices: £1 835 for PFC Sigma, £1 150 for AGC Biomet, £1 676 for Nexgen, £1 294 for Genesis 2, and £1 325 for Triathlon (Lewis P. NHS SupplyChain. Personal Communication).

Unit costs of the operating theatre for a primary TKR (£2 022) and a hospital bed day (£332) were based on data from a recent RCT carried out in the UK [24]. The unit costs of revisions (£8 429) recognised that more resources are used than for primary surgery [24].

### Statistical analysis to provide input parameters for the cost-effectiveness model

#### QOL after primary TKR and revision

We estimated QOL following primary TKR according to prosthesis brand using linear regression to adjust for observed differences in pre-operative patient and provider characteristics between the comparison groups. We adjusted for the following differences in case mix: age, sex, comorbidities, BMI, disability, ASA grade, IMD, patella replacement, surgical position, pre-operative EQ-5D-3L and OKS scores. To avoid attributing differences arising from surgeon or hospital factors to prosthesis brand, we also adjusted for provider characteristics (surgeon experience [senior surgeon or not], and hospital type [specialist treatment centre or general hospital]).

We applied fractional polynomials to continuous variables where they provided an improvement in model fit over linear or quadratic functions [25]. We then predicted post-operative QOL by prosthesis brand separately for men and women in three different age groups (60, 70 and 80 years). QOL in the year during which revision TKR took place, and in subsequent years after revision was predicted according to age and sex using linear regression.

Of the 53 126 patients included in the QOL analysis 17% were missing post-operative PROMs. Most other data items were missing for less than 10% of the sample. Multiple imputation using chained equations was applied to pre- and post-operative data to impute missing responses [26]. Twenty imputations were undertaken and results across these imputations were combined by Rubin’s rules [27].

#### Rate of revision and re-revision

Annual revision rates were predicted from NJR data for each prosthesis brand after adjusting for differences between the comparison groups. We adjusted for differences in case mix (age, sex, ASA grade, BMI, patella replacement, antibiotic cement) and provider characteristics (surgeon experience, and hospital type). We used a restricted cubic spline regression model to capture the underlying variation in revision rates over time, without imposing a pre-specified relationship, and to allow extrapolation of revision rates beyond the observation period [28]. To meet the requirements of the Markov model, re-revision rates were estimated from HES data with a piece-wise constant survival regression model which differentiated revision rates at only one time point: the first year versus all subsequent years.

### Cost-effectiveness analysis

For men and women aged 60, 70 and 80, the cost-effectiveness model reported revision rates, costs related to TKR, and QALYs for patients with the average pre-operative characteristics for each subgroup. A recommended annual discount rate of 3.5% was applied to both costs and outcomes to reflect societal time preferences [29].

We report the incremental costs per QALY, and the probability that each prosthesis brand is the most cost-effective. We recognised sampling uncertainty in the estimation of the model parameters by undertaking a probabilistic analysis. Model results are reported after averaging across 1 000 simulations in which each model parameter was sampled from the appropriate probability distribution.

For each brand of TKR, we calculated the net monetary benefit by multiplying total lifetime QALYs by society’s willingness-to-pay for a QALY gain and subtracting from this the total lifetime cost. The calculation was repeated with alternative levels of willingness-to-pay for a QALY gain (from £0 to £50 000). We calculated cost-effectiveness acceptability frontiers to report the brand with the highest *mean* net monetary benefit (most cost-effective on average) and the proportion of simulations for which this brand had the highest net monetary benefit (recognising sampling variation), at different levels of willingness-to-pay for a QALY gain [30].

#### Sensitivity analyses

We tested whether the results were robust to alternative assumptions about post-operative QOL, revision rates and costs across brands. First, we assumed that differences in post-operative QOL between prosthesis brands were only maintained for one year after TKR, rather than until a revision or death. Second, we considered an alternative form of linear regression model to estimate post-operative QOL across TKR brands by including interaction terms between prosthesis brand and both age and sex. Third, in the linear regression model that estimated the effect of brand on post-operative QOL, rather than including a single continuous variable for the baseline EQ-5D-3L, we specified categorical variables for each health state dimension. Fourth, we considered an alternative form of the regression model used for the prediction of revision rates by using a piece-wise constant rather than a restricted cubic spline hazard function. Fifth, we applied the same prosthesis cost to each brand.

The Markov model was built in Excel; statistical analysis to parameterize the model was undertaken in STATA version 11.

The study is exempt UK NREC approval as it involved analysis of existing datasets of anonymised data for service evaluation. Approvals for the use of HES data were obtained as part of the standard Hospital Episode Statistics approval process.

## Results

### Patient characteristics before the total knee replacement

The pre-operative characteristics of the sample of 53 126 patients who were used for estimating the effect of prosthesis brand on post-operative QOL were very similar across brands (Table 1). However, patients receiving the PFC Sigma and the Triathlon were more likely to have had the TKR at an independent sector treatment centre and by a consultant, than those receiving the other three brands.

**Table 1:** Characteristics of 53 126 patients according to knee prosthesis brands with multiple imputation of missing data.

	Prosthesis Brand				
	PFC Sigma	AGC Biomet	Nexgen	Genesis 2	Triathlon
No. of patients	13 635	5 005	3 364	4 187	3 585
Mean (SD) age (years)	69.9 (7.3)	70.5 (7.3)	69.8 (7.3)	70.1 (7.3)	69.9 (7.3)
Proportion Male (%)	45	44	45	44	43
Most deprived fifth (%)	20	16	21	22	23
Two or more comorbidities (%)	30	29	30	29	28
ASA grade 3 or higher (%)	16	17	18	16	18
Mean (SD) BMI (kg/m^2^)	31.3 (5.5)	31.4 (5.7)	31.4 (5.6)	31.3 (5.5)	31.0 (5.6)
Operation at Independent sector treatment centre (%)	10	2	0	1	14
Operation by consultant (%)	84	79	76	77	82
Pre-operative mean (SD) OKS	18.5 (7.6)	19.0 (7.7)	18.6 (7.4)	19.0 (7.6)	18.8 (7.7)
Pre-operative mean (SD) EQ-5D-3L index	0.38 (0.31)	0.41 (0.31)	0.39 (0.31)	0.41 (0.31)	0.40 (0.31)

### Initial outcomes and costs after primary TKR

After adjustment for pre-operative characteristics, the mean OKS, and EQ-5D-3L score six months after the primary TKR was highest in those receiving a Nexgen and lowest in those receiving a Genesis 2 (Table 2). Revision rates at 5 and 10 years were lowest with the Nexgen and PFC Sigma brands and highest with the AGC Biomet (Table 2). Initial costs were lowest with the AGC Biomet and highest with the PFC Sigma.

**Table 2:** Initial QOL, OKS, initial cost and revision rates after primary TKR, according to brand with adjustment for pre-operative differences in case mix, for men and women aged 70.

	Prosthesis Brand				
	PFC Sigma	AGC Biomet	Nexgen	Genesis 2	Triathlon
	**Men aged 70**				
Post-operative mean EQ-5D-3L index	0.73	0.72	0.74	0.71	0.72
Post-operative mean OKS score	35.4	35.4	36.0	34.2	35.3
Mean cost of primary replacement (£)	5 414	4 574	5 233	5 229	5 006
5-year revision rate	2.1%	2.8%	2.1%	2.4%	2.5%
10-year revision rate	3.1%	4.2%	3.1%	3.7%	3.8%
	**Women aged 70**				
Post-operative mean EQ-5D-3L index	0.72	0.71	0.73	0.70	0.71
Post-operative mean OKS score	33.9	33.8	34.4	32.6	33.7
Mean cost of primary replacement (£)	5 491	4 651	5 311	5 306	5 084
5-year revision rate	1.6%	2.2%	1.6%	1.9%	2.0%
10-year revision rate	2.5%	3.3%	2.5%	2.9%	3.0%

### Cost-effectiveness analysis

**Fig 2:**
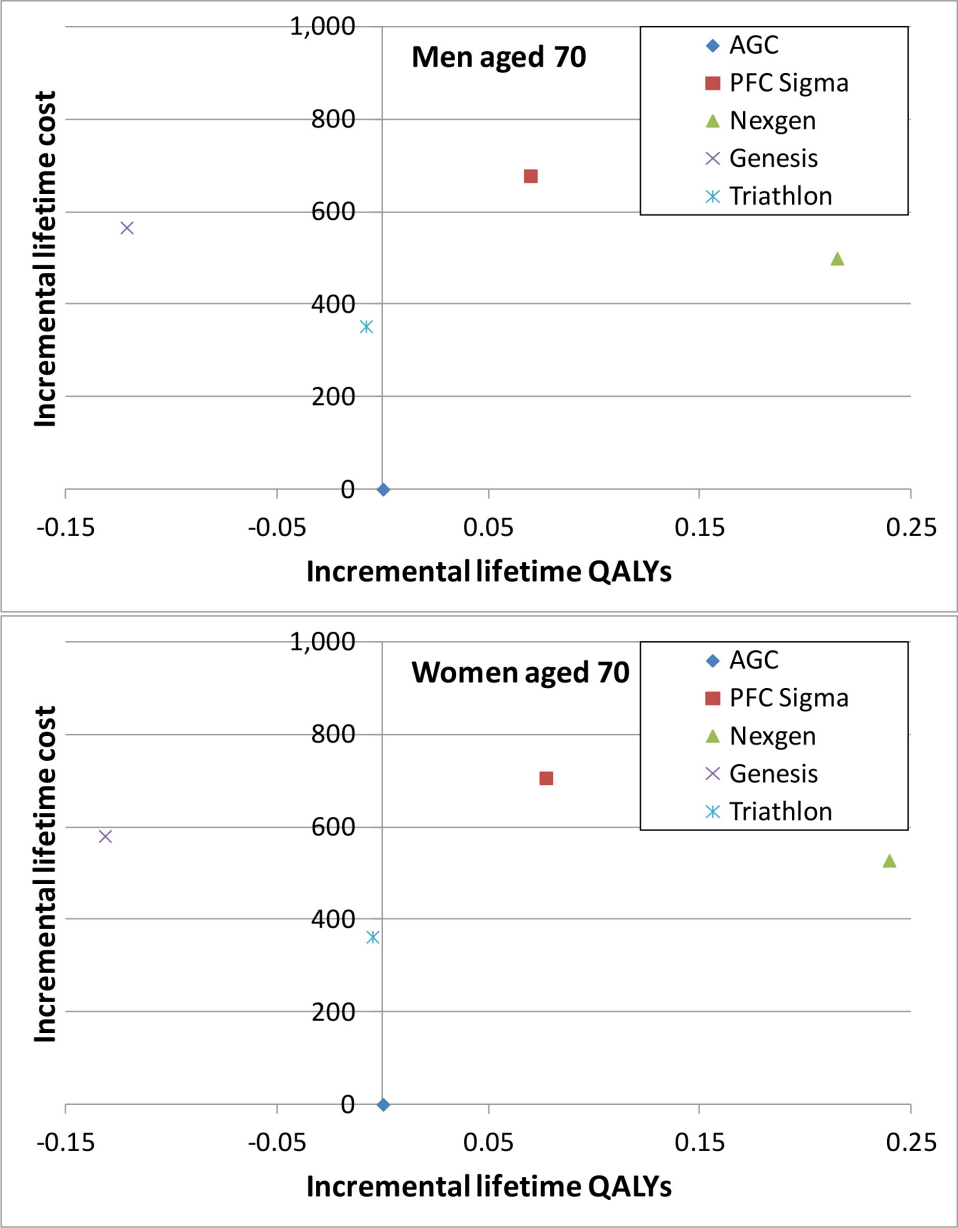
Cost-effectiveness planes for men and women aged 70. The axis represents differences in lifetime QALYs and cost compared to those of AGC, the prosthesis brand with the lowest lifetime cost.

For both men and in women, the average lifetime costs were lowest in those who received an AGC Biomet and highest in those who received a PFC Sigma (Table 3). Patients who had a Nexgen prosthesis had the highest expected quality-adjusted life expectancy (QALYs). The Genesis 2 and Triathlon brands were “dominated” by the AGC Biomet as they had lower average lifetime QALYs and higher mean costs (Fig 2). The PFC Sigma was dominated by the Nexgen. For 70-year old men and women the incremental cost-effectiveness ratio for receiving a Nexgen compared to an AGC Biomet prosthesis was approximately £2 300 per QALY.

**Table 3:** Mean lifetime costs, lifetime QALYs, and proportion of patients undergoing revision and net monetary benefit for men and women aged 70.

	**Prosthesis Brand**				
	PFC Sigma	AGC Biomet	Nexgen	Genesis 2	Triathlon
	**Men aged 70**				
Lifetime proportion revised	3.9%	5.1%	3.8%	4.5%	4.6%
Mean lifetime cost (£)	5 900	5 226	5 721	5 799	5 600
Mean lifetime QALYs	7.57	7.50	7.72	7.38	7.50
Net monetary benefit at £20,000 per QALY (£)	145 553	144 818	148 658	141 867	144 343
Incremental Cost-effectiveness ratio (ICER)	dominated	base	2 284	dominated	dominated
	**Women aged 70**				
Lifetime proportion revised	3.3%	4.5%	3.3%	3.9%	4.0%
Mean lifetime cost (£)	5 876	5 166	5 696	5 756	5 553
Mean lifetime QALYs	8.27	8.20	8.44	8.06	8.19
Net monetary benefit at £20,000 per QALY (£)	159 556	158 819	163 012	155 498	158 289
Incremental Cost-effectiveness ratio (ICER)	dominated	base	2 244	dominated	dominated

**Fig 3:**
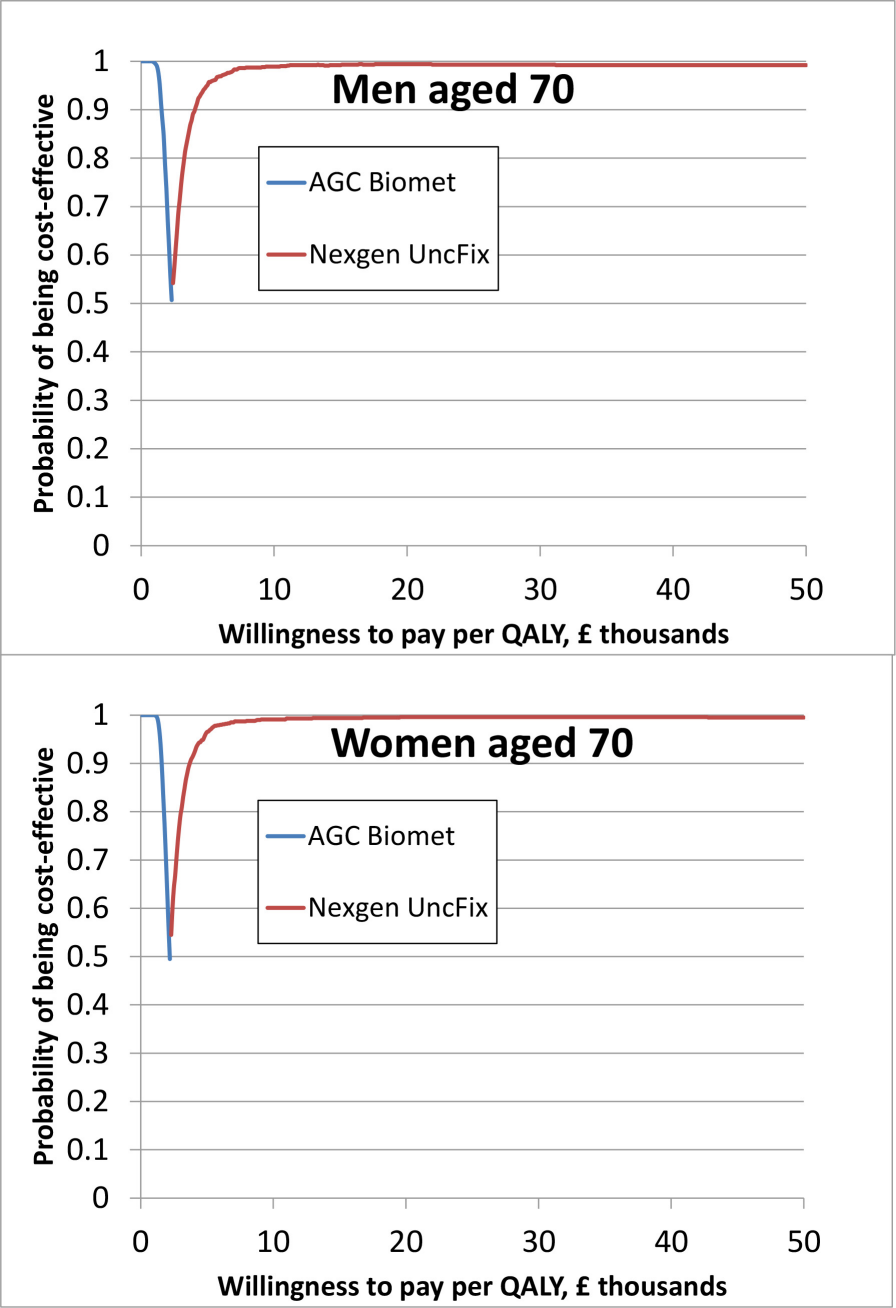
Cost-effectiveness acceptability frontiers, 70 year olds, base case. Only the result of the prosthesis brand with the highest average net monetary benefit for a given willingness-to-pay threshold is presented in the figure.

Similarly, the cost-effectiveness results in Table 3 and the acceptability frontiers in Fig 3 demonstrate that for patients aged 70, the Nexgen is highly likely to be the most cost-effective brand, or in other words, had the highest net monetary benefit from the vast majority of the 1 000 model simulations. If the societal willingness-to-pay per QALY exceeds £10 000, the probability that the Nexgen is the most cost-effective prosthesis brand exceeds 98% for men and women aged 70. A similar pattern of results was observed for men and women age 60 and 80 and the Nexgen is therefore also highly likely to be the most cost-effective brand for these age groups.

### Sensitivity analyses

The finding that the Nexgen was the most cost-effective brand was robust to alternative assumptions (Table 4). When differences in post-operative QOL between brands were assumed to be maintained for only one year, and when the linear regression analysis estimating the effect of brand on post-operative QOL included interactions between brand, age and sex, the results were somewhat more uncertain. However, the probability that the Nexgen is the most cost-effective prosthesis is always 75% or above for each subgroup at a willingness-to-pay threshold of £20 000 per QALY.

**Table 4:** Proportion of 1 000 simulations that a knee prosthesis brand gave the highest net monetary benefit at £20 000 per QALY for men and for women aged 70 in the base case and sensitivity analyses.

	Proportion of 1 000 simulations in which the prosthesis was the most cost-effective (achieved the highest net monetary benefit) by brand				
	PFC Sigma	AGC Biomet	Nexgen	Genesis 2	Triathlon
**Scenario**	**Men aged 70**				
Base case	0·006	0	0·994	0	0
Differences in QOL between brands only maintained in first year after TKR	0·003	0·131	0·843	0	0·023
Interaction between brand, age and sex included in regression model for prediction of post-operative QOL	0·013	0·099	0·825	0	0·063
Pre-operative QOL specified using dummies for EQ-5D responses	0·003	0	0·997	0	0
Piece-wise constant hazard function for prediction of revision rate	0·018	0·011	0·965	0	0·006
Same prosthesis cost for all brands	0·002	0	0·998	0	0
	**Women aged 70**				
Base case	0·003	0	0·996	0	0·001
Differences in QOL between brands only maintained in first year after TKR	0	0·226	0·750	0	0·024
Interaction between brand, age and sex included in regression model for prediction of post-operative QOL	0·003	0·003	0·994	0	0
Pre-operative QOL specified using dummies for EQ-5D responses	0·003	0	0·996	0	0·001
Piece-wise constant hazard function for prediction of revision rate	0·022	0·007	0·967	0	0·004
Same prosthesis cost for all brands	0·006	0	0·994	0	0

## Discussion

Our cost-effectiveness analysis found that the Nexgen was the most cost-effective cemented, unconstrained, fixed prosthesis brand for patients with osteoarthritis who have a primary TKR. Selection of the Nexgen over the other four most commonly used brands would generate better outcomes for patients at low additional costs. These results have implications for many countries as the five included prosthesis brands represent the majority of prostheses implanted for example in Sweden, Finland, Denmark, Australia, New Zealand and the US [9,31–35].

This is the first study to compare the cost-effectiveness of commonly used brands of knee prosthesis. Previous comparisons across brands only reported revision rates or symptom severity and post-operative QOL [9,10,12,36]. We synthesised evidence on revision rates, post-operative QOL and costs and found that relatively small differences in post-operative QOL are the key determinants of relative cost-effectiveness. For example, patients who received the Nexgen had the highest post-operative QOL but similar 5-year and 10-year revision rates compared to the PFC Sigma, the market leader in the UK. In our previous cost-effectiveness analysis of alternative types of total hip replacement we also found that the relative effect on post-operative QOL was the key driver of cost-effectiveness [15]. This highlights the importance of having data from a large national PROMs programme linked to the NJR.

Whilst other joint registries have a longer follow-up than the NJR, the value of older data is limited given the recognised gradual improvement of joint replacement outcome in the last decade [37] and the ongoing introduction of new knee prostheses. Moreover, we were able to exploit the linkage of PROMs data to both HES and the NJR which allowed us to quantify small but potentially important differences in post-operative QOL between brands while carefully adjusting for differences in patient and provider characteristics across brands.

It should be acknowledged that residual confounding is a potential explanation of small differences between treatment groups in observational studies irrespective of their size. However, in estimating the effects of prosthesis brand on QOL and revision rates, we adjusted for a wide range of case mix variables including pre-operative symptom severity, comorbidity and BMI. Also, our sensitivity analysis demonstrated that even with alternative specifications of the linear regression model used to adjust for pre-operative case mix differences, the Nexgen remained the most cost-effective prosthesis. Lastly, the choice of prosthesis brand depends on the hospital and the surgeons providing the treatment rather than on the pre-operative characteristics of the patient, which reduces the risk of residual confounding even further. Two thirds of hospitals in England use a single brand for the majority of patients because it is advantageous for hospitals to use one brand from a single supplier for most patients as that would allow them to negotiate a discounted price. We did not undertake hierarchical modelling to estimate post-operative QOL and consequently there is a possibility that we under-estimated uncertainty around differences in post-operative QOL and in the resultant estimates of cost-effectiveness.

Post-operative QOL was based on data collected 6 months after TKR. There is some evidence of relatively small further gains in QOL beyond 6 months [38]. However, this increase is small and unlikely to influence the relative differences between the brands. In addition, our sensitivity analysis demonstrated that the cost-effectiveness results are robust to variations in the duration that the differences in post-operative QOL between prosthesis brands are maintained.

Our analysis did not explore the reasons for differences in QOL outcomes or revision rates between brands. The prosthesis design factors influencing revision rates and QOL are complex and multifactorial. Nevertheless, Joint registries show improvement in design over time. Our findings are consistent with a previous report of the superiority of QOL outcomes with the Nexgen prosthesis [12].

We obtained information on the costs of the prosthesis brands from NHS SupplyChain, an organisation that provides NHS hospitals with equipment and materials they need for patient care. Using these national costs has the advantage they represent the actual costs paid in the UK rather than a list price. We expect that in other countries, existing market mechanisms would produce a similar price pattern across the brands. However sensitivity analysis in which each prosthesis brand was assigned the same cost suggest the results are not sensitive to variation in prosthesis costs.

In conclusion, our study found that the initial and lifetime costs of the Nexgen prosthesis are lower than the PFC Sigma, the market leading brand, and that patients who received the Nexgen knee prosthesis have higher lifetime QALYs compared to four commonly used alternative brands of unconstrained, fixed prosthesis. The Nexgen prosthesis is therefore the most cost-effective brand, across all patient subgroups. These results suggest that the Nexgen should be considered as a first choice prosthesis for patients with osteoarthritis who require a TKR.

## Supporting Information

S1 Information (PDF)

## Abbreviations

ASA: American Society of Anesthesiologists
BMI: Body Mass Index
HES: Hospital Episode Statistics
IMD: Index of Multiple Deprivation
LOS: Length of Stay
NHS: National Health Service
NJR: National Joint Registry of England, Wales and Northern Ireland
OKS: Oxford Knee Score
PROMs: Patient Reported Outcome Measures
QALY: Quality Adjusted Life Year
QOL: health related quality of life
TKR: Total Knee Replacement
RCT: Randomised Controlled Trial
