# Supplementary material for: Cost-Effectiveness of Five Commonly Used Prosthesis Brands for Total Knee Replacement in the UK: A Study Using the NJR Dataset
Source: PLoS One. 2016 Mar 4;11(3):e0150074. doi: 10.1371/journal.pone.0150074 (PMC4778929; doi:10.1371/journal.pone.0150074)
Supplement: S1 Information — (PDF) [file pone.0150074.s001.pdf]

Technical Appendix

Cost-effectiveness of five commonly used prosthesis brands for total knee replacement in the UK

Contents

Overview

Section 1: Data sources and linkage

Section 2: Multiple imputation of missing data

Section 3: Analysis of QOL after primary TKR and before and after revision TKR

Section 4: Quantifying LOS following primary and revision TKR

Section 5: Analysis of primary revision rates

Section 6: Estimation of re-revision rates

Section 7: Analysis of mortality following TKR

Section 8: Parameterisation of sampling uncertainty

Section 9: Results for 60 and 80 year olds

Section 10: Details on sensitivity analyses and resulting CEAFs

Section 1: Data sources and linkage

Data sources

The National Health Service (NHS) has collected outcome data on all total knee replacement (TKR); total hip replacement; groin hernia surgery; and varicose vein removal undertaken in England since implementing the Patient Reported Outcome Measures Programme (PROMs) programme in April 2009.[1] Pre-operative data is collected prior to surgery and a post-operative questionnaire is sent six months after surgery to patients returning the pre-operative questionnaire. Response rates for the pre-operative questionnaire for TKR are 90%.[2] We accessed data on 211 459 patients who underwent TKR between Aug 2008 and January 2012. This included 11 894 respondents to the pilot survey[3] which preceded the PROMs programme. Eighty-six percent of these patients had returned a post-operation questionnaire by the end of July 2012.

The National Joint Registry of England, Wales and Northern Ireland (NJR) began collecting data on joint replacement operations in 2003.[4] We received data on TKR from inception to March 2012 for patients aged 55 to 84 inclusive, with a diagnosis of osteoarthritis, undergoing surgery in an NHS hospital or treatment centre or funded by the NHS (n = 265 910).

Hospital Episode Statistics (HES) is the administrative database of NHS funded inpatient hospital stays.[5] Data includes length of stay (LOS); Index of Multiple Deprivation (IMD, a measure of socio-economic status);[6] International Classification of Disease (ICD-9) diagnosis codes;[7] Office of Population, Censuses and Surveys (OPCS) procedure codes, and date of death where appropriate.[8] We received HES data from April 1998 to March 2012, and extracted data on 756,484 primary TKRs and 78 279 revision procedures using OPCS codes. From the primary, cemented TKR data we excluded patients aged under 55 or over 84, and patients whose TKR was undertaken for reasons other than osteoarthritis (based on ICD-9 codes) leaving 616 938 patients. Primary TKR and subsequent revisions on the same knee were linked using pseudo-anonymised patient identification codes and laterality indicators.

Linkage of data sets and application of exclusion criteria to the linked data

The NHS information centre was able to link 70% of TKR records in PROMS to the corresponding HES episode and 55% of PROMs records to the procedure entry in the NJR.[9] All PROMs records successfully linked to a record in the NJR were also linked to a record in HES. For 60% of the records in the linked PROMs-HES-NJR data, component data from the NJR were available, allowing unambiguous assignment of the brand of prosthesis. The impact of exclusions on the linked PROMs-HES-NJR data is illustrated in Figure 1 below.

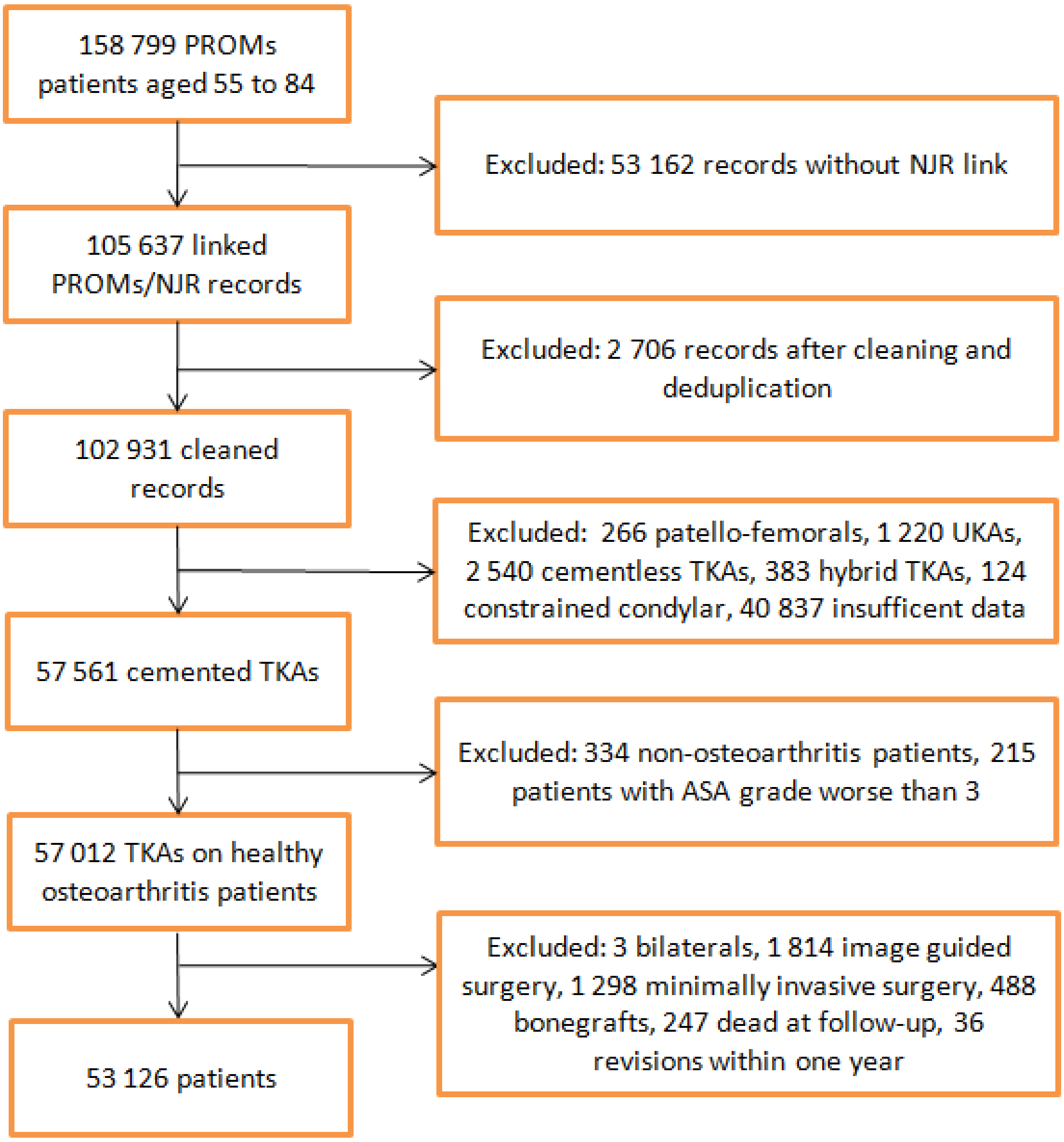

Figure 1: flowchart illustrating exclusions prior to the analysis of linked PROMs-HES-NJR data

Section 2: Multiple imputation of missing data

Missing pre-operative data in the linked PROMs-HES-NJR dataset was generally below 10% with the exception of BMI (67%). Missing post-operative data ranged from 17-24% and arose predominantly from missing questionnaires. Missing data was imputed using Chained Equations (ICE command in Stata).[10]

The MI equations included all variables which entered the regression models for LOS and QOL, including age-brand and sex-brand interaction terms which were included in a sensitivity analysis of post-operative QOL. Pre- and post-operative EQ-5D-3L responses were specified using dichotomous variables to indicate a level two or three response to each of the five dimensions. This approach utilised the information available from partially complete EQ-5D-3L responses. Pre-operative EQ-5D-3L tariff scores, specified using fractional polynomials as they appeared in the regression models for QOL and LOS, were also included. We further included ethnicity, total number of diagnosis (ICD) codes in HES, duration of symptoms, measures of overall health (such as the EQ-5D-3L Visual Analogue Scale), and measures of satisfaction with surgery. Ordered categorical data, including quintiles of IMD and EQ-5D visual analogue scale responses, were imputed using ordered logistic regression. Oxford knee scores were imputed using predicted mean matching to allow for the truncation and skew of the distribution. Post-operative LOS and the total number of ICD-9 codes in HES were log transformed to reduce skew. Twenty imputations were undertaken.

QOL and LOS before and after imputation are illustrated in Table 1 below. Post-operative EQ-5D-3L index scores were lower after imputation by a similar amount across all five TKR brands (0.01 QALYs).

**TKR brand**	**Post-op EQ-5D-3L index**		**Post-op OKS score**		**LOS**	
	**Complete case**	**Mean of 20 imputations**	**Complete case**	**Mean of 20 imputations**	**Complete case**	**Mean of 20 imputations**
PFC Sigma	0.713	0.702	34.59	33.73	5.11	5.11
AGC Biomet	0.715	0.705	34.19	33.92	4.98	4.98
Nexgen	0.729	0.721	34.72	34.44	5.16	5.16
Genesis 2	0.705	0.693	33.21	32.86	5.22	5.22
Triathlon	0.714	0.705	34.21	33.94	5.37	5.37

Table 1 - key outcome variables by TKR brand before and after imputation

Section 3: Analysis of QOL after primary TKR and before and after revision TKR

Linear Regression was used to estimate post-operative QOL by brand after primary TKR and adjusting for differences in patient and provider characteristics. The analysis adjusted for ASA grade, BMI, disability, type of hospital, comorbidities, IMD, EQ-5D-3L pre-score, OHS pre-score, patella replacement, surgical position (medial parapatellar or not), age and sex. Model fit, was assessed by Akaike's Information Criteria (AIC).[11] Fractional polynomials were used to specify the relationship of the dependent variable to pre-operative EQ-5D-3L tariff and pre-operative OKS.[12] Fractional polynomial forms of age and BMI were investigated, but rejected in favour of a linear specification of BMI and a quadratic specification of age, as the latter, simpler specifications yielded regression models with equivalent fit. The model was used to predict QOL for the six subgroups (men and women aged 60, 70 and 80) and for each of the five TKR brands assuming mean values from the relevant subgroup population for the dependent variables.

The analysis indicated a gain of 0.016 QALYs ($p = 0.002$) and 0.48 OKS points ($p = 0.006$) for Nexgen prostheses compared to prostheses not identified amongst the top five brands; differences were not significant for the remaining top four brands. These findings proved robust to alternative specifications of the regression model including one in which pre-operative QOL entered the model using dichotomous variables to specify responses to each of the five dimensions of the questionnaire. A head to head comparison of the Nexgen and PFC Sigma using linear regression generated similar results (a gain of 0.016 QALYs ($p = 0.002$) for the Nexgen) as did analysis of the complete case data (0.015 QALYs, $p = 0.002$). Inclusion of interactions between age and TKR brand and sex and TKR brand did not lead to a significant improvement in model fit. However, this model was retained for a sensitivity analysis.

Extrapolation of QOL gains

The health state tariffs for each health state in the model were reduced with each cycle to reflect the impact of age on QOL. The adjustment utilised the regression model of the impact of age on QOL reported by Brazier and Ara, and based on a large sample of UK data.[13] In this model the impact of age is curvilinear; QOL drops at an increasing rate with advancing age. The yearly decrement for patients aged 70 is 0.004QALYs.

Health State tariffs during and after revision

The PROMs data included 6 769 patients undergoing a revision TKR of which 6 128 were 55 or over. Of these patients, 5761 had a pre-operative EQ-5D-3L tariff score and 3 912 had a post-operative EQ-5D-3L tariff score. Linear regression of scores *prior* to surgery generated QOL tariffs for the Markov state 'Revision' as a function of age and sex. Likewise, the QOL tariff for the Markov state 'revised TKR' was parameterised as a function of age and sex using EQ-5D-3L tariff scores *following* revision surgery.

Section 4: Quantifying LOS following primary and revision TKR

Linear regression was used to estimate differences in LOS after primary TKR by brand after investigation revealed the data was only moderately skewed. We adjusted for ASA grade, BMI, home environment (living alone or not), disability, type of hospital, comorbidities, IMD, EQ-5D-3L pre-score, OHS pre-score, patella replacement, surgical position (medial parapatellar or not), age and sex. After adjustment for patient and provider characteristics LOS varied by 0.5 days across brands with the AGC Biomet associated with the shortest LOS. We found very similar results using a Generalised Linear Model which assumed a Gamma distribution for LOS. The linear regression model was used to predict LOS for the six subgroups (men and women aged 60, 70 and 80) and for each of the five TKR brands assuming mean values from the relevant subgroup population for the dependent variables.

Linear regression was also used to model LOS after revision TKR as a function of age and sex. The model utilised the data on revision TKR in the linked PROMs-HES-NJR data.

Section 5: Analysis of primary revision rates

There are multiple causes of TKR revisions including infection, surgical errors during primary TKR and failure or loosening of components. The relative importance of these causes varies with time since surgery. The resulting overall hazard function is poorly approximated by a simple monotonic function with respect to time. Consequently, we chose to use restricted cubic splines to model the log cumulative hazard.[14] This technique is well documented and provides a flexible approach to modelling event data.

A restricted cubic spline model was fitted to the NJR data. It adjusted for ASA grade (classified as 1, 2 or other), BMI (classified as <30; 30 to 35; >35), patella replacement, surgical position (medial parapatellar or not); use of antibiotic cement; surgeon grade (consultant level or not), hospital type (general or specialist orthopaedic centre), age and sex. Patients with missing BMI data were assumed to have a BMI below 30. Model fit was judged by AIC. Optimal fit was obtained using the hazard scale with four degrees of freedom (three internal knots) for the baseline hazard and allowing a time dependent effect of age (one degree of freedom). The model allowed prediction of revision rates within the observed period after adjustment for casemix and extrapolation of revision rates beyond the observed period.

Section 6: Estimation of re-revision rates

OPCS codes identified 78 279 patients in HES undergoing a revision TKR between 1st April 1998 and 31st March 2012. 7111 patients aged under 55 and 4434 patients with no data on laterality were excluded. Revisions occurring on the same knee were linked using pseudo-anonymised patient identifiers and laterality codes. We assumed the earliest revision recorded on a knee was the first revision and the next procedure was a *re-revision*. Further operations were not considered. The resulting data had a mean follow-up time of 4.2 years and consisted of 52 660 patients of which 9 978 underwent *re-revisions*. Preliminary analysis showed a higher *re-revision* rate in the first year following surgery. The data was modelled using a piece-wise constant hazard function with a single boundary at one year.[15] The resulting hazard was constant with respect to time after the first year but varied with patient age. The overall revision risk fell as patients aged. Probabilities over a range of ages are tabulated below (table 2). The probability of *re-revision* in the first year was applied to patients in the revision state and an event resulted in patients remaining in this state to undergo a further revision in the following cycle. The probability of *re-revision* in subsequent years was applied to patients in the revised TKR state and an event resulted in transition to the revision state.

	Probability of failure of revised prosthesis (%)			
	Men		Women	
Age	First year after surgery	Years 2+ after surgery	First year after surgery	Years 2+ after surgery
60	16.93	3.41	12.52	2.47
65	16.17	2.82	11.95	2.04
70	15.44	2.33	11.40	1.69
75	14.75	1.92	10.87	1.39
80	14.08	1.59	10.37	1.15
85	13.44	1.31	9.89	0.95
90	12.82	1.08	9.43	0.78

Table 2 – probability of failure after years after revision TKR

Section 7: Analysis of mortality following TKR

Operative mortality was estimated as 30day mortality using logistic regression and adjusting for age and sex. We analysed HES data on primary TKR from April 1998 to March 2012 for patients 55 to 84 with a diagnosis of osteoarthritis after excluding patients who underwent revision within 30days to avoid double counting (n = 597 595).

All cause mortality was derived from UK life tables after adjustment to reflect the lower mortality observed in patients undergoing elective TKR and total hip replacement compared with age and sex matched cohorts.[16,17] Data on patients undergoing primary TKR in HES from April 1998 to March 2012, aged between 55 and 84, and with a primary diagnosis of osteoarthritis (n = 598 970) was split into 900 cohorts defined by age at operation, sex and calendar year. Mortality rates were determined for the second to the twelfth year after surgery for each cohort. The rates were paired with the rate derived from the UK life table[18] for the cohort matched on age, sex and calendar year. Data from the first year was discarded to avoid double counting operative mortality during primary TKR. Tobit regression was used to model the ratio of the two rates as a function of age, sex and years after primary surgery. Tobit regression was selected to allow for the truncation of the distribution of the ratio at zero. The ratio predicted by the function was used to adjust mortality probabilities from the UK life table before applying them to the cohorts in the model.

Section 8: Parameterisation of sampling uncertainty

The Markov model was fully probabilistic. Sampling uncertainty around each of the parameters was incorporated by specifying each parameter as a random variable with mean and variance derived from the regression model used in its estimation. Each coefficient in the respective regression models was assumed to follow a normal distribution, and correlation of the uncertainty around coefficient estimates was captured by applying the Cholesky decomposition of the covariance-correlation matrix.[19] As a result, transition parameters in the Markov model were assumed to be drawn from log-normal distributions, and health state QOL tariffs and LOS were assumed to be drawn from normal distributions. Whilst in principal this allowed for the possibility of generating QOL tariffs or LOS beyond feasible values, in practice standard errors were small enough to render this virtually impossible.

Section 9: Results for 60 and 80 year olds

Tables 3 and 4 report the model parameters and model results for men and women aged 60 and 80. Figures 2 to 4 provide prosthesis survival curves, cost-effectiveness planes and Cost-effectiveness Acceptability Frontiers (CEAFs) for men and women aged 60 and 80.

Brand	PFC Sigma	AGC Biomet	Nexgen	Genesis 2	Triathlon
			Men aged 60		
EQ-5D-3L index	0.685	0.680	0.698	0.668	0.679
Initial Cost (£)	5 268	4 427	5 087	5 083	4 860
5-year revision rate	3.6%	4.8%	3.6%	4.2%	4.4%
10-year revision rate	5.9%	7.9%	5.9%	6.9%	7.1%
			Women aged 60		
EQ-5D-3L index	0.664	0.659	0.678	0.647	0.658
Initial Cost (£)	5 350	4 510	5 170	5 165	4.943
5-year revision rate	2.9%	3.8%	2.9%	3.4%	3.5%
10-year revision rate	4.7%	6.3%	4.7%	5.5%	5.7%
			Men aged 80		
EQ-5D-3L index	0.730	0.725	0.744	0.713	0.724
Initial Cost (£)	5 860	5 020	5 680	5 675	5 453
5-year revision rate	1.2%	1.6%	1.2%	1.4%	1.4%
10-year revision rate	1.6%	2.2%	1.6%	1.9%	2.0%
			Women aged 80		
EQ-5D-3L index	0.716	0.711	0.729	0.699	0.710
Initial Cost (£)	5 945	5 104	5 764	5 760	5 537
5-year revision rate	0.9%	1.3%	0.9%	1.1%	1.1%
10-year revision rate	1.3%	1.7%	1.3%	1.5%	1.6%

Table 3 - Initial cost, QOL after primary TKR and survival at five and ten years after adjusting for casemix for men and women aged 60 and 80.

Brand	PFC Sigma	AGC Biomet	Nexgen	Genesis 2	Triathlon
			Men aged 60		
Proportion revised	10.0%	13.2%	10.0%	11.6%	12.0%
Mean Cost (£)	6 457	6 002	6 281	6 462	6 300
Mean QALYs	9.28	9.15	9.47	9.02	9.15
NMB at £20,000 per QALY (£)	179 078	177 004	183 064	173 890	176 746
			Women aged 60		
Proportion revised	8.8%	11.6%	8.8%	10.2%	10.5%
Mean Cost (£)	6 281	5 751	6 108	6 254	6 063
Mean QALYs	9.84	9.71	10.04	9.56	9.71
NMB at £20,000 per QALY (£)	190 456	188 491	194 744	184 861	188 188
			Men aged 80		
Proportion revised	1.5%	2.1%	1.5%	1.8%	1.9%
Mean Cost (£)	6 066	5 297	5 886	5 916	5 705
Mean QALYs	5.15	5.11	5.25	5.03	5.10
NMB at £20,000 per QALY (£)	96 951	96 850	99 073	94 593	96 364
			Women aged 80		
Proportion revised	1.3%	1.7%	1.3%	1.5%	1.6%
Mean Cost (£)	6 108	5 324	5 928	5 951	5 735
Mean QALYs	5.68	5.64	5.79	5.54	5.63
NMB at £20,000 per QALY (£)	107 517	107 448	109 898	104 884	106 837

Table 4 - Mean lifetime costs, lifetime QALYs, proportion of patients undergoing revision and NMB for men and women aged 60 and 80

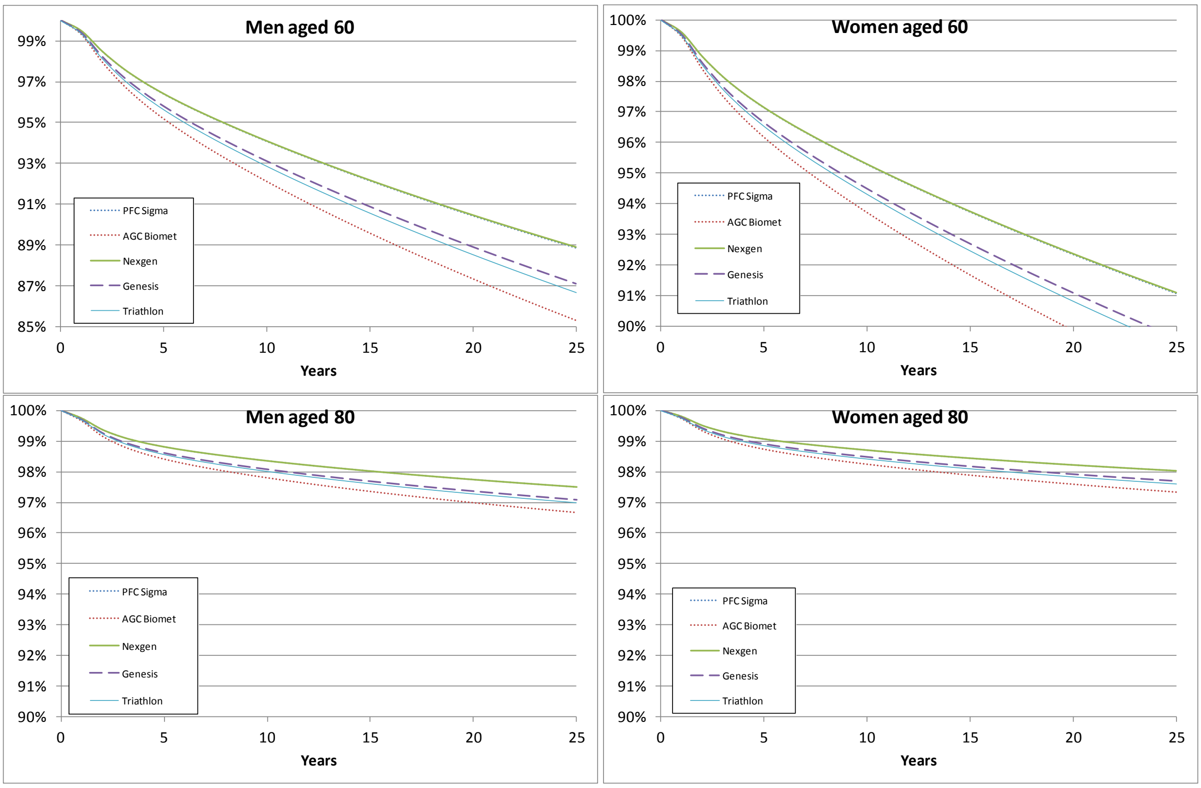

Figure 2 - Predicted prosthesis survival in men and women aged 60 and 80 after adjusting for casemix

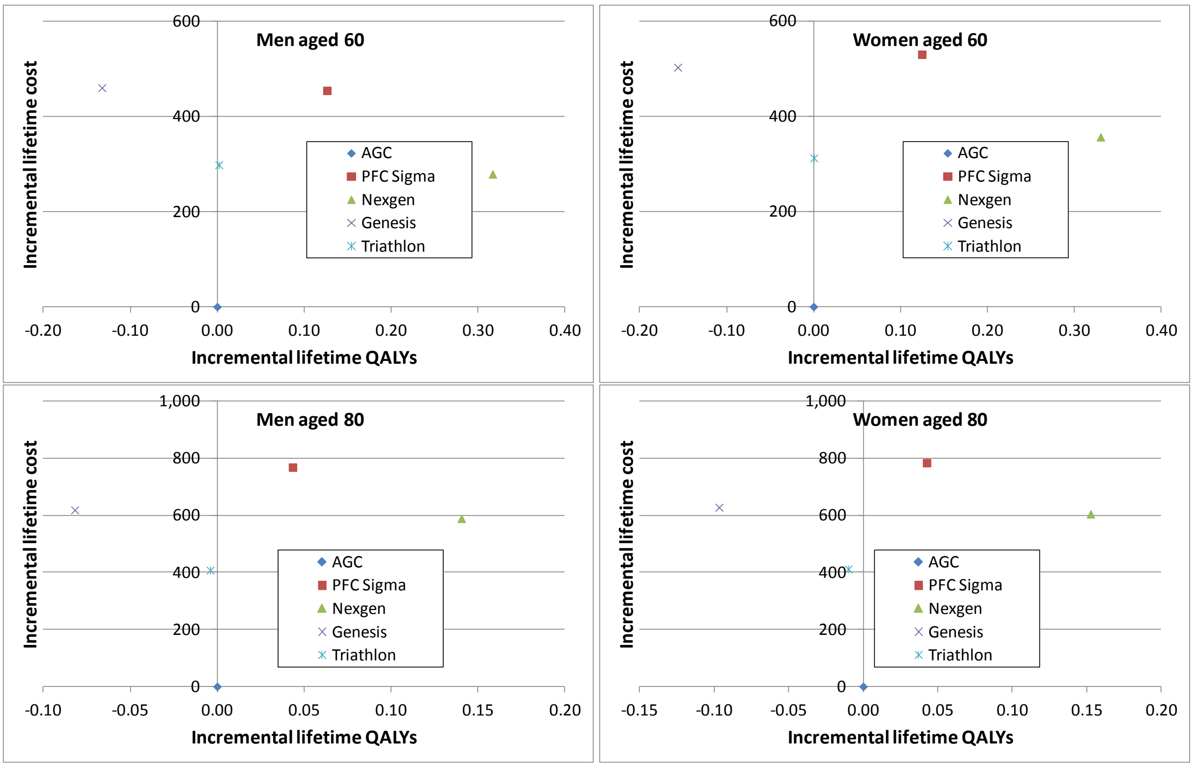

Figure 3 - Cost-effectiveness planes for men and women aged 60 and 80

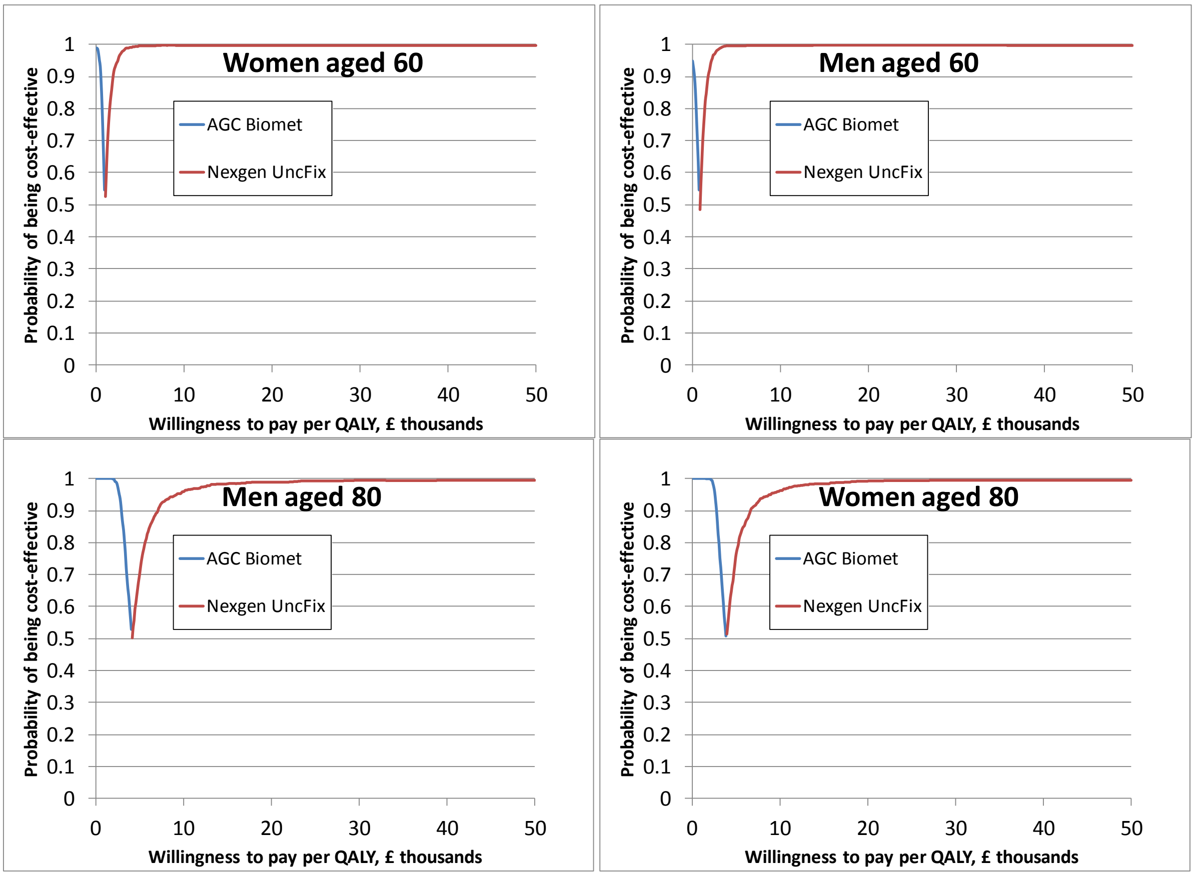

Figure 4 - Cost-effectiveness acceptability frontiers (CEAFs) for men and women aged 60 and 80

Section 10: details of the sensitivity analyses

Figures 5 to 9 illustrate the CEAFs obtained in each of the six subgroups after running the following sensitivity analyses: analysis of QOL after primary TKR using an alternative linear regression model; assuming the same cost for all prosthesis brands; extrapolating revision rates using a Piece-wise Constant model; allowing interactions between variables specifying subgroup and brand in the regression model for post-operative QOL; assuming differences in QOL by TKR brand are maintained for one year only. Further details of the methods are provided below.

Analysis of QOL after primary TKR using an alternative linear regression model

In the base case analysis, the regression model used to estimate post-operative QOL by brand included pre-operative EQ-5D-3L specified as the tariff score. In a sensitivity analysis we applied an alternative regression model for post-operative QOL in which pre-operative QOL entered the model using dichotomous variables to identify a response at level 2 or level 3 on each of the five dimensions of the instrument (ten dummies in total).

Assuming the same cost for all prosthesis brands

To investigate the impact of primary prosthesis costs on the results we ran a sensitivity analysis in which all prostheses were assumed to cost the same as the PFC Sigma.

Extrapolating revision rates using a Piece-wise Constant model

The Restricted Cubic Spline model used to extrapolate revision rates by brand in the base case, was replaced with a Piece-wise Constant model.[20] The model specified intervals at the end of years 1,2,3,4 and 5, effectively allowing the baseline hazard to change at these intervals according to the data. Dummies specified for the years 1 through 5 were interacted with each of the five brands (25 interaction terms in total). The resulting model allowed hazard rates to vary each year for the first five years and by brand. Revision rates beyond five years were assumed constant across brands and constant with respect to time. Revision rates still varied according to age and sex. The model included the same dependent variables as those specified in the Restricted Cubic Spline model used in the base case.

Allowing interactions between subgroup and brand in the estimation of post-operative QOL

The regression model used in the base case to predict post-operative QOL by brand did not include interaction terms for age and sex with the dichotomous variables identifying brand, as their inclusion did not lead to an improvement in model fit. A regression model which included these interactions was used to predict post-operative QOL in a sensitivity analysis.

Assuming differences in QOL by TKR brand are maintained for one year only

Differences in QOL in the primary TKR state according to brand were applied for the first cycle (year) only. In subsequent cycles the QOL tariff for patients with a PFC Sigma (for the relevant subgroup) was applied to all patients in the primary TKR state regardless of brand fitted. The adjustment to QOL tariffs in each health state due to aging remained as in the base case.

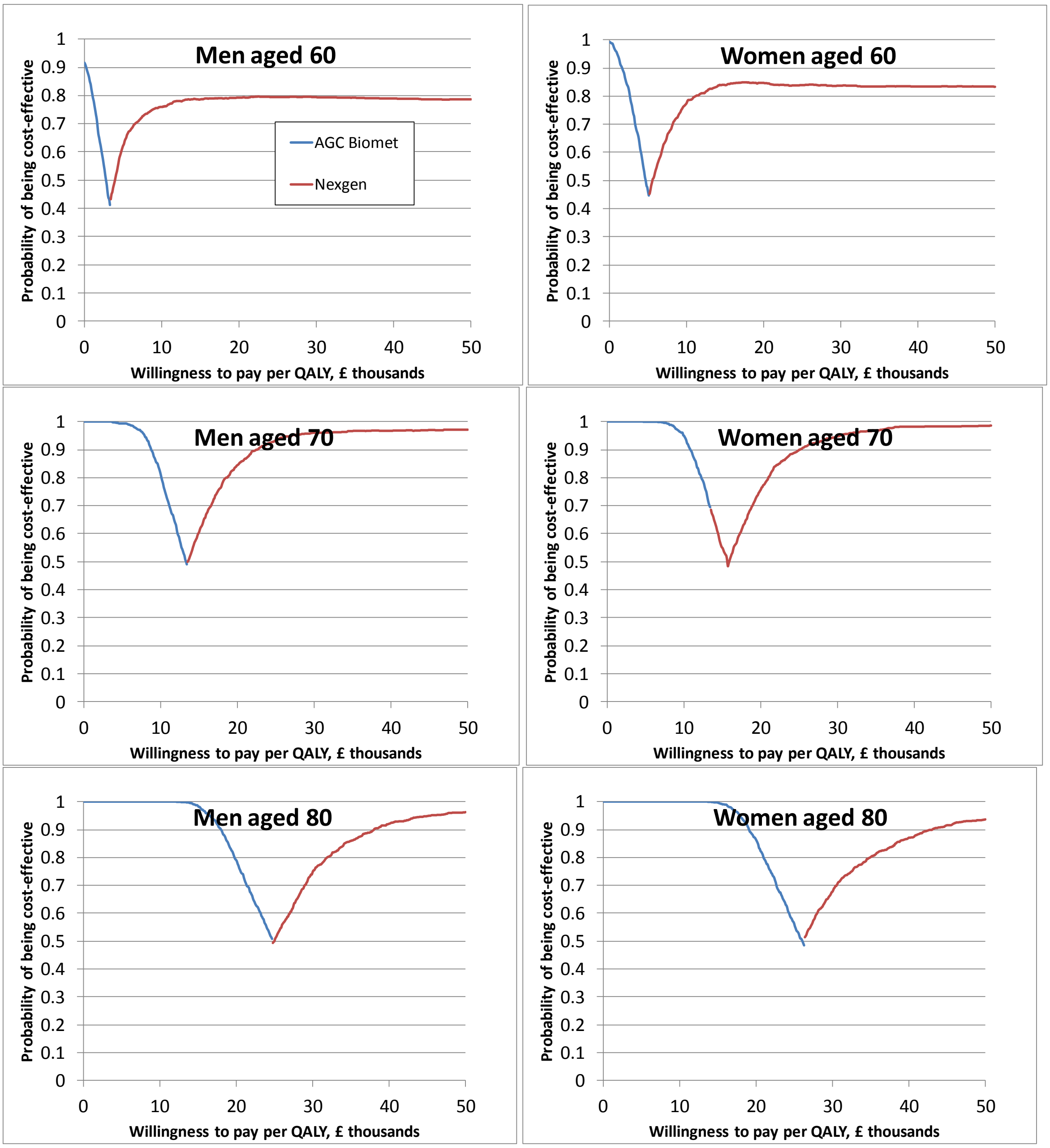

Figure 5 - CEAFs by subgroup assuming differences in QOL by TKR brand are maintained for 1 year post surgery

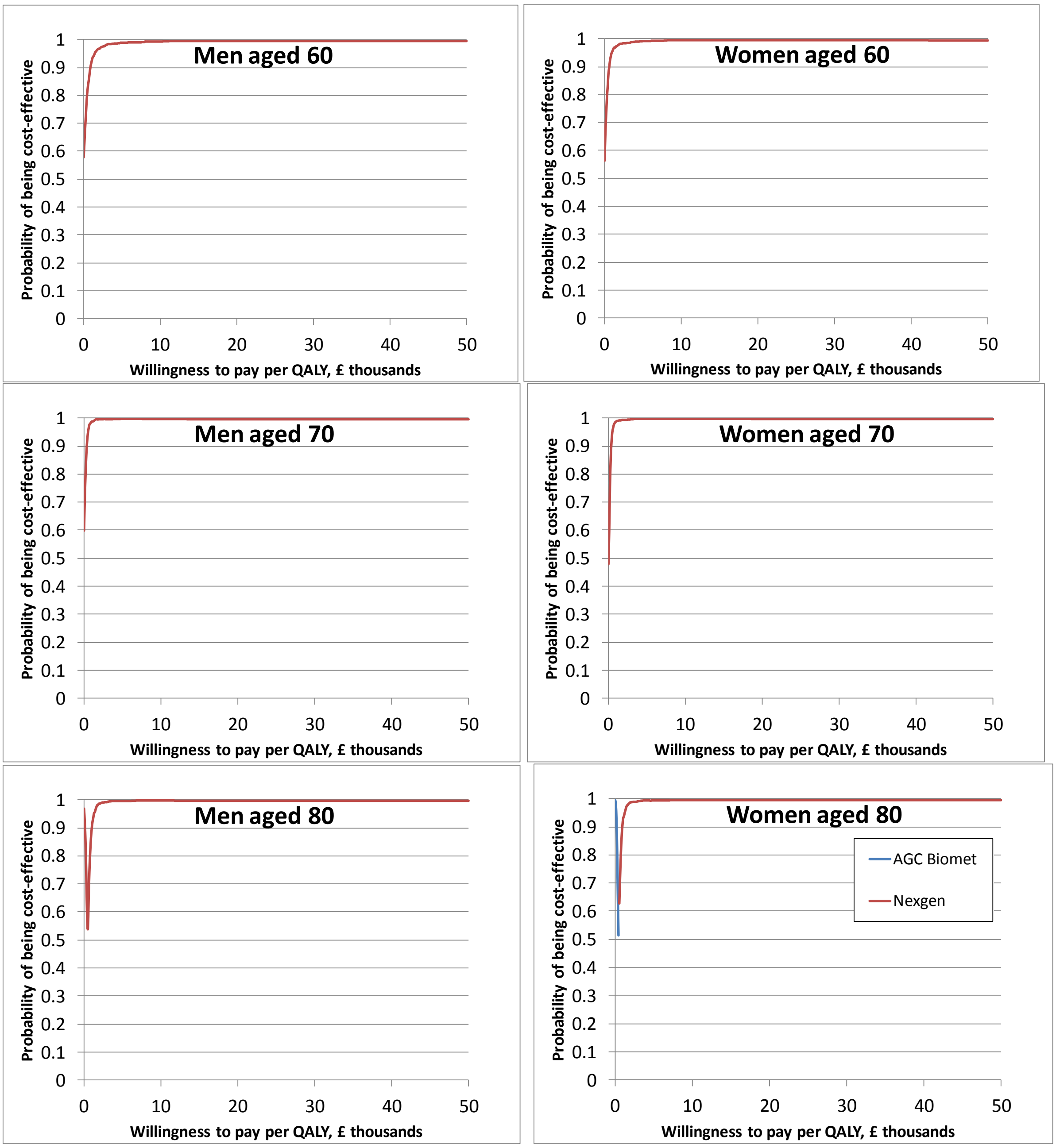

Figure 6 - CEAFs by subgroup after assuming the same cost for all brands of prosthesis.

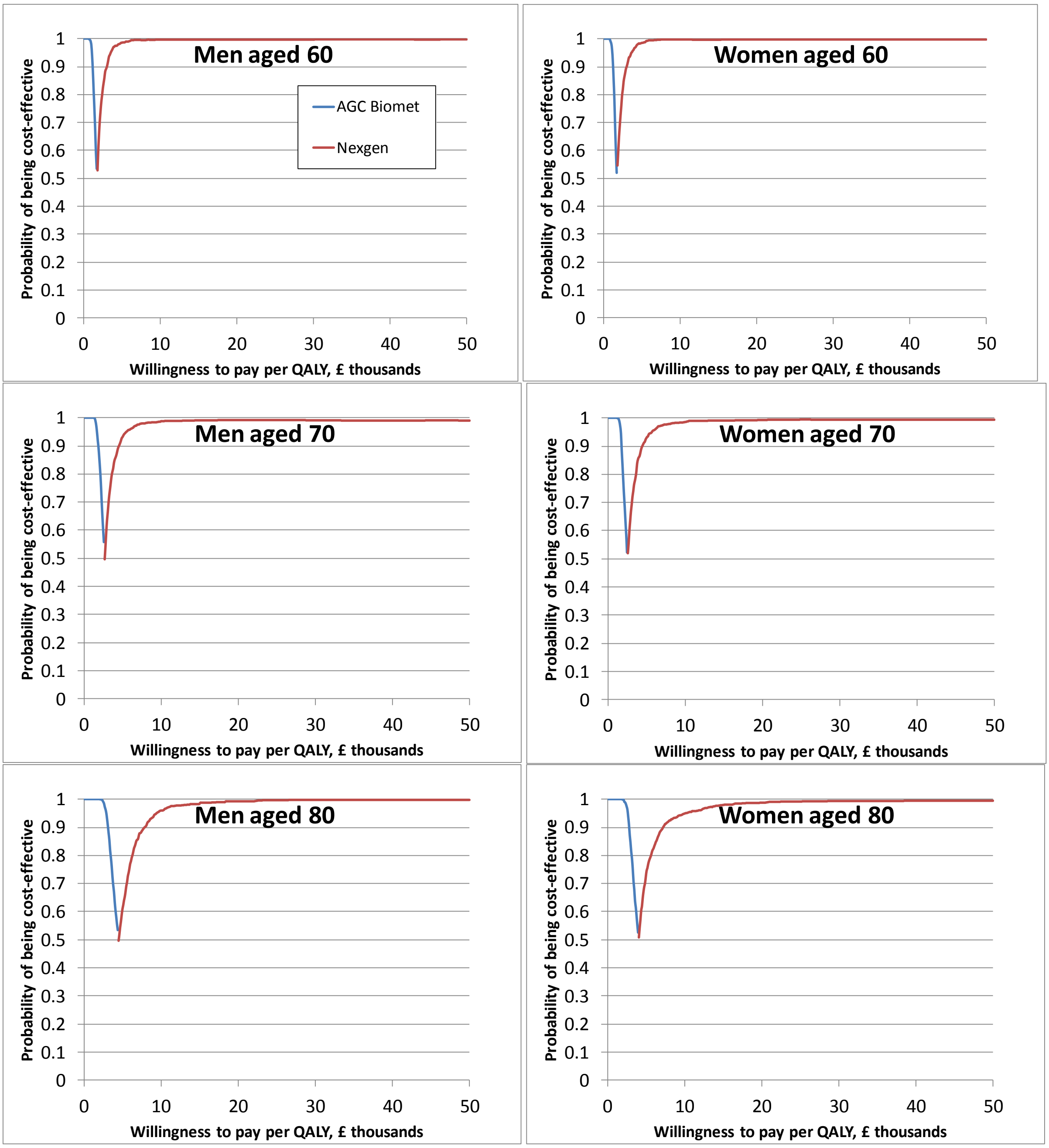

Figure 7 - CEAFs by subgroup after extrapolation of revision rates using a Piece-wise Constant survival model.

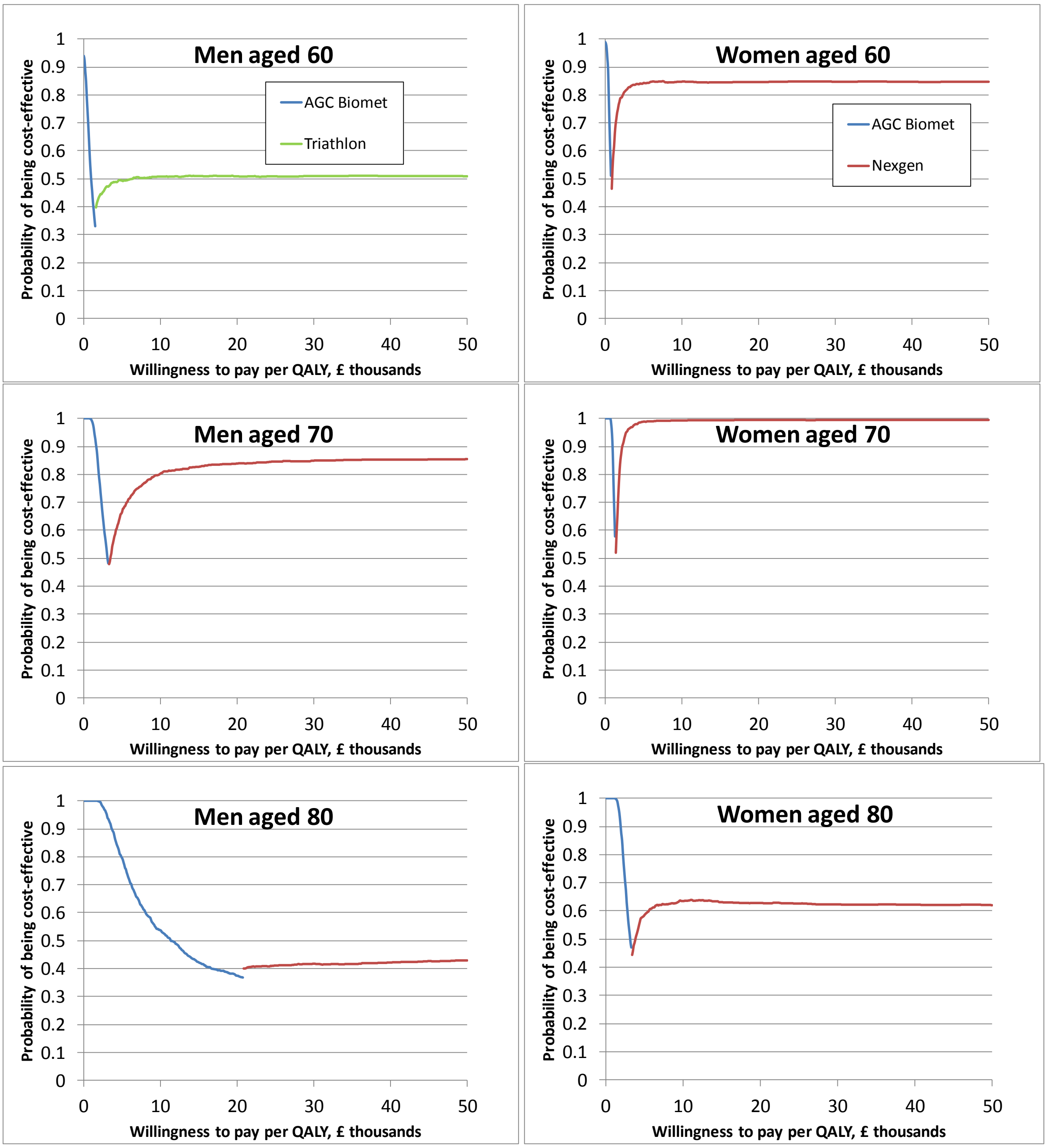

Figure 8 - CEAFs by subgroup after allowing interaction between subgroup and brand in the model used to predict post-operative QOL

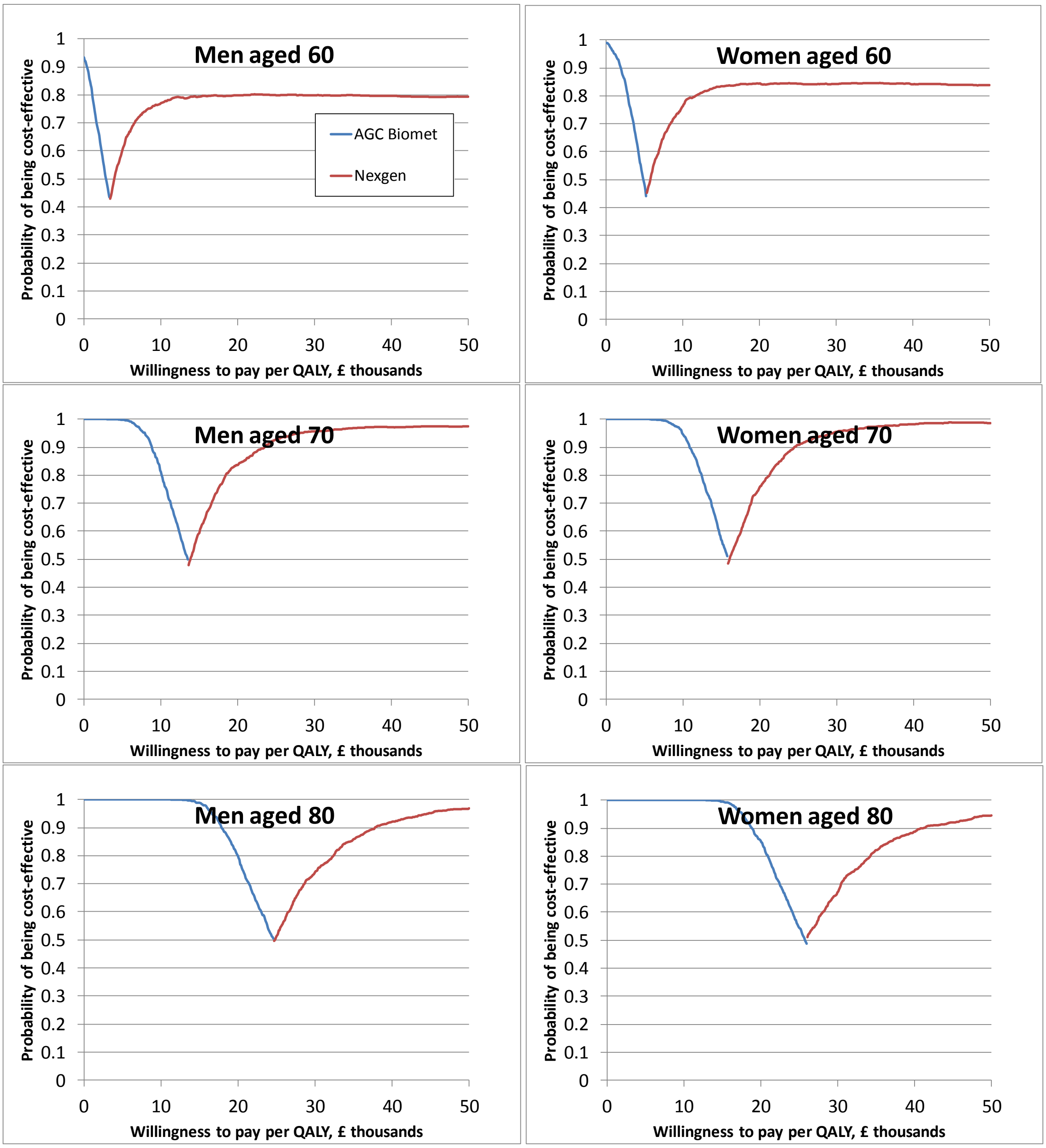

Figure 9 - CEAFs by subgroup after assuming that differences in post-operative QOL between brands persist only for the first year following surgery

References

1. Ousey K, Cook L. Understandin patient reported outcome measures (PROMs). *British Journal of Community Nursing* 2011; **16**: 80–2.

2. Health and Social Care Information Centre. Provisional Monthly Patient reported Outcome Measures (PROMs) in England - April 2012 to March 2013, November 2013 release: csv Data Pack. Available at: http://www.hscic.gov.uk/searchcatalogue?productid=12572&q=title%3a%22Provisional+Monthly+Patient%22&sort=Relevance&size=10&page=1#top. Accessed 8th May 2014.

3. Chard J, Kuczawski M, van der Meulen J. A report comparing Independent Sector Treatment Centres and NHS providers: POiS Audit Steering Committee, Clinical Effectiveness Unit, The Royal College of Surgeons of England; 2011.

4. National Joint Registry, Northgate Information Solutions (UK) ltd, Hemel Hempstead, Hertfordshire, UK. www.njrcentre.org.uk Accessed 8th May 2014.

5. Health and Social Care Information Centre, Hospital Episode Statistics. Available at: http://www.hscic.gov.uk/hes. Accessed 8th May 2014.

6. Noble M, Mclennan D, Wilkinson K, et al. The English Indices of Deprivation 2007. Wetherby: Department of Communities and Local Government; 2007.

7. International Classification of Diseases: World Health Organisation.

8. Classification of Surgical Operations and Procedures - 4th revision (opcs4) codes: Office of Population, Censuses and Surveys.

9. Health and Social Care Information Centre. Provisional Monthly Patient reported Outcome Measures (PROMs) in England. A guide to PROMs methodology. Available at: http://www.hscic.gov.uk/media/1537/A-Guide-to-PROMs-Methodology/pdf/PROMS_Guide_v5.pdf. Accessed 8th May 2014.

10. White IR, Royston P, Wood AM. Multiple imputation using chained equations: Issues and guidance for practice. *Stat Med* 2011; **30**: 377–99.

11. Akaike H. A new look at the statistical model identification. *Automatic Control, IEEE T Automat Cont* 1974; **19**(6): 716–723.

12. Royston P, Ambler G, Sauerbrei W. The use of fractional polynomials to model continuous risk variables in epidemiology. *Int J Epidemiol* 1999; **28**(5): 964–974.

13. Ara R, Brazier JE. Populating an economic model with health state utility values: moving toward better practice. *Value in Health* 2010; **13**(5): 509–518.

14. Royston P, Lambert PC. Flexible parametric survival analysis using Stata: beyond the Cox model. *Stata Press*, 2011.

15. Colvert RE, Boardman TJ. Estimation in the piece-wise constant hazard rate model. *Commun Stat-Theory M* 1976; **5**(11): 1013–1029.

16. Schrøder HM, Kristensen PW, Petersen MB, Nielsen PT. Patient survival after total knee arthroplasty: 5-year data in 926 patients. *Acta Orthop* 1998; **69**(1): 35–38.

17. Lie SA, Engesaeter LB, Havelin LI, Gjessing HK, Vollset SE. Mortality after total hip replacement: 0-10-Year follow-up of 39,543 patients in the Norwegian Arthroplasty Register. *Acta Orthop Scand 2000*; **71**: 19–27.

18. United Kingdom, Interim Life Tables, 1980-82 to 2007-09: Office for National Statistics

19. Briggs AH, Claxton K, Sculpher MJ. *Decision modelling for health economic evaluation*. Oxford university press, 2006.

20. Colvert RE, Boardman TJ. Estimation in the piece-wise constant hazard rate model. *Commun Stat-Theory M* 1976; **5**(11): 1013–1029
